# Evaluation of efficacy and safety of glucokinase activators—a systematic review and meta-analysis

**DOI:** 10.3389/fendo.2023.1175198

**Published:** 2023-05-08

**Authors:** Wenjia Yang, Han Wu, Xiaoling Cai, Chu Lin, Ruoyang Jiao, Linong Ji

**Affiliations:** Department of Endocrinology and Metabolism, Peking University People’s Hospital, Beijing, China

**Keywords:** glucokinase activators, efficacy, safety, diabetes, hypoglycemia

## Abstract

**Aims:**

Glucokinase activators (GKAs) promote the activity of glucokinase (GK) and is under development for the treatment of diabetes. The efficacy and safety of GKAs require evaluation.

**Methods:**

This meta-analysis included randomized controlled trials (RCTs) with a duration of at least 12 weeks conducted in patients with diabetes. The primary objective of this meta-analysis was the difference of hemoglobin A1c (HbA1c) change from baseline to study end between GKA groups and placebo groups. Risk of hypoglycemia and laboratory indicators were also evaluated. Weighted mean differences (WMDs) and 95% confidence intervals (CIs) were calculated for the continuous outcomes, and odds ratios (ORs) and 95% CI were calculated for the risk of hypoglycemia.

**Results:**

Data from 13 RCTs with 2,748 participants treated with GKAs and 2,681 control participants were analyzed. In type 2 diabetes, the level of HbA1c decreased greater in patients with GKA treatment compared with placebo (WMD = -0.339%, 95% CI -0.524 to -0.154%, P < 0.001). The OR comparing GKA versus placebo was 1.448 for risk of hypoglycemia (95% CI 0.808 to 2.596, P = 0.214). The WMD comparing GKA versus placebo was 0.322 mmol/L for triglyceride (TG) levels (95% CI 0.136 to 0.508 mmol/L, P = 0.001). When stratified by drug type, selectivity, and study duration, a significant difference was found between groups. In type 1 diabetes, the result of HbA1c change and lipid indicators showed no significant difference between the TPP399 group and the placebo group.

**Conclusions:**

In patients with type 2 diabetes, GKA treatment was associated with a better glycemic control but a significant elevation in TG concentration in general. The efficacy and safety varied with drug type and selectivity.

**Systematic review registration:**

International Prospective Register of Systematic Reviews, identifier CRD42022378342.

## Introduction

Diabetes is a complex, chronic illness requiring continuous medical care. In 2021, approximately 537 million people were living with diabetes worldwide, of whom 90%–95% were diagnosed with type 2 diabetes and 1.2 million were diagnosed with type 1 diabetes (1). The management of these populations is particularly challenging. Despite that novel drug therapeutic strategies acting with different mechanisms of actions have been developed continuously, unmet needs still exist in clinical practice.

Glucokinase (GK) is an enzyme that functions as a glucose sensor, which plays a critical role in glucose homeostasis. GK regulates glucose-stimulated insulin secretion (GSIS) and intrinsic glucagon release in pancreatic cells. In the liver, GK promotes hepatic glucose uptake, glycogen synthesis, and storage (2, 3). Based on the hypothesis that control over GK activity might affect glucose homeostasis, GK activators (GKAs) emerged as a distinct glucose-lowering option in the management of diabetes. Recently, one GKA was approved for treatment of type 2 diabetes in China (4) and several are in the late stage of clinical development.

GKAs are small molecules that bind to an allosteric site on GK with effects of promoting GK activation. These GKAs can be classified according to the site of action: pancreatic and hepatic dual-acting GKAs and hepatoselective GKAs (5). So far, several GKAs have been developed and tested for clinical application purposes, whereas different efficacies and safeties were shown in these agents. The main concerns with use of GKAs focused on the increased risk of worsening of dyslipidemia, hypoglycemia, and liver damage (5, 6). In a randomized controlled trial (RCT), MK-0941 was found to be associated with hemoglobin A1c (HbA1c) reduction of -0.5% to -0.8% and a significant increased incidence of hypoglycemia and hypertriglyceridemia by 30 weeks in patients with treatment of insulin (7). Two RCTs evaluating AZD1656 demonstrated different results in HbA1c reduction, while an increased risk of hypertriglyceridemia was observed (8, 9). However, recently phase 3 RCTs conducted in drug-naïve or metformin-treated type 2 diabetes patients showed that dorzagliatin could improve HbA1c for a duration of 24 weeks without significant increased risk of hypoglycemia. However, a consistent increased level of liver enzymes, serum triglyceride, and serum uric acid (SUA) was observed in the study groups that received dorzagliatin treatment (10, 11). Due to the inconsistencies shown in clinical trials with regard to efficacies and side effects, it is feasible and necessary to make a comprehensive meta-analysis to evaluate the efficacy and safety of GKA treatment.

## Materials and methods

### Systematic review protocol

The Preferred Reporting Items for Systematic Reviews and Meta-Analyses statement was used to conduct this meta-analysis (12). The study protocol is available in the International Prospective Register of Systematic Reviews (registration no. CRD42022378342)

### Data sources and search strategy

We searched electronic databases including PubMed, EMBASE, and the Cochrane Central Register of Controlled Trials last updated on 01/12/2022. Two investigators (HW and WY) independently searched for clinicals trials of GKAs. The search terms were as follows: RCTs; GK; GKA; piragliatin; RO4389620; dorzagliatin; HMS5552; globalagliatin; SY-004; LY2608204; MK-0941; AZD1656; PB-201; PF-04937319; PF-04991532; AMG 151; ARRY-403; TTP399; GKI-399; AZD 6370; TMG123. The search strategies were as follows: (1) RCTs and GK; (2) RCTs and GKA; (3) every GKA was added respectively as the collateral searching term addition to RCTs. The detail of the search strategy is shown in **Supplementary Table 1**.

### Selection criteria and data extraction

The criteria for including the studies were as follows: (1) RCTs conducted in diabetes patients (both type 1 diabetes and type 2 diabetes); (2) RCTs with GKAs and placebo control in different treatment arms; (3) since the primary outcome of this meta-analysis was to evaluate the efficacy of GKAs, RCTs with study duration ≥12 weeks were selected; (4) RCTs with available data of glucose control with or without safety outcomes. The exclusion criteria were as follows: (1) non-RCTs; (2) RCTs with study duration<12 weeks; (3) RCTs that did not report efficacy or safety outcomes.

We removed the duplicates and screened the remaining articles at the title and abstract levels according to the predetermined inclusion and exclusion criteria for possible inclusions. The process of search and selection was performed by two independent blinded investigators (HW and WY) with Covidence software. If either investigator considered a study potentially eligible, we further obtained and screened the full text. We invited a third investigator (CL) to join the discussion and resolved discrepancies by consensus.

Two investigators (WY and HW) independently performed the data extraction from each publication using a standardized form: publication data, baseline characteristics of the study population (sample size, sex, age, body mass index [BMI], baseline body weight), baseline laboratory indicators (HbA1c, fasting plasma glucose [FPG], postprandial plasma glucose [PPG], fasting insulin [FINS], homeostasis model assessment of insulin resistance [HOMA-IR], homeostasis model assessment-β [HOMA-β], total cholesterol [TCHO], triglyceride [TG], low-density lipoprotein cholesterol [LDL-C], high-density lipoprotein cholesterol [HDL-C], alanine transaminase [ALT], aspartate aminotransferase [AST], serum uric acid [SUA], estimated glomerular filtration rate [eGFR]), incidence of hypoglycemia, duration of follow-up, description of the GKA and control interventions, and changes of laboratory indicators from baseline to study end point. Disagreements or discrepancies were resolved by discussion between the two investigators and a third investigator (XC).

The PICOTS (population, interventions, comparators, outcomes, timing, and setting) criteria of this meta-analysis were summarized as follows (**Table 1**):

**Table 1:** The PICOTS for the evaluation of efficacy and safety of glucokinase activators.

Population	Patients with type 2 diabetes patients or type 1 diabetes
Interventions	Glucokinase activators, including piragliatin; RO4389620; dorzagliatin; HMS5552; globalagliatin; SY-004; LY2608204; MK-0941; AZD1656; PB-201; PF-04937319; PF-04991532; AMG 151; ARRY-403; TTP399; GKI-399; AZD 6370; and TMG123.
Comparisons	Placebo
Outcomes	Primary outcome: The difference of HbA1c change from baseline between the GKA group and placebo group. Secondary outcomes: (i) The difference of FPG, PPG, FINS, HOMA-IR, HOMA-β, TCHO, TG, LDL-C, HDL-C, ALT, AST, SUA, and eGFR change from baseline between the GKA group and placebo group. (ii) The risk of hypoglycemia comparing the GKA group with the placebo group.
Time	Published before 01/12/2022
Study design	Randomized controlled trials

PICOTS, population, interventions, comparators, outcomes, timing, and setting; GKA, glucokinase activator; HbA1c, hemoglobin A1c; FPG, fasting plasma glucose; PPG, postprandial plasma glucose; FINS, fasting insulin; HOMA-IR, homeostasis model assessment of insulin resistance; HOMA-β, homeostasis model assessment -β; TCHO, total cholesterol; TG, triglyceride; LDL-C, low-density lipoprotein cholesterol; HDL-C, high-density lipoprotein cholesterol; ALT, alanine transaminase; AST, aspartate aminotransferase; SUA, serum uric acid; eGFR, estimated glomerular filtration rate.

### Assessment of methodological quality

Two investigators (WY and HW) independently assessed the quality of each included studies using a modified Cochrane risk-of-bias instrument that includes response options of “definitely or probably yes” (assigned as low risk of bias) or “definitely or probably no” (assigned as high risk of bias) (13). Each study is judged on seven items: sequence generation, allocation concealment, blinding of participants and personnel, blinding of outcome assessors, incomplete outcome data, selective outcome reporting, and other sources of bias.

### Data synthesis and analysis

Weighted mean differences (WMDs) and 95% confidence intervals (CIs) were calculated for continuous measures. We used mean changes from baseline and standard deviations (SDs) extracted from published data when reported. We calculated mean changes from baseline by subtracting baseline means from outcome means and calculated change SDs from baseline by using methods outlined in the Cochrane Handbook when authors reported results as measures before and after intervention (14). When SDs were missing, we estimated them from standard errors or confidence intervals. Odds ratios (ORs) and 95% CI were calculated for odds of hypoglycemia. For studies with multiple intervention arms, we selected the groups relevant to the purpose of our study. Then, we combined groups to create a single pairwise comparison recommended by the Cochrane Handbook (14).

Between-study heterogeneity was assessed using the Q test and *Ι (* 2) statistic, with significance set at P < 0.05; heterogeneity was considered low, moderate, substantial, or considerable for estimated *I (* 2) values of 0%–40%, 30%–60%, 50%–90%, and 75%–100%, respectively. The random-effect model was used in this meta-analysis. To interpret the level of heterogeneity, we also calculated prediction intervals, as these intervals could reflect the variation in treatment effects over different settings, including what effect is to be expected in patients in the future.

Meta-regression analysis was performed to evaluate the association between change in HbA1c level, risk of hypoglycemia, change in TG concentration, and clinical features. Potential confounding factors, including age, sex, BMI, and duration of diabetes, were adjusted by using multivariable meta-regression analysis. A P value <0.05 was considered statistically significant.

Funnel plot and Egger’s test were used to assess publication bias. Statistical analyses were conducted with Review Manager, version 5.4 (Nordic Cochrane Centre, Copenhagen, Denmark), and STATA statistical software package, version 17 (Stata Corp, College Station, TX, United States).

## Results

### Overall analysis

**Figure 1:**
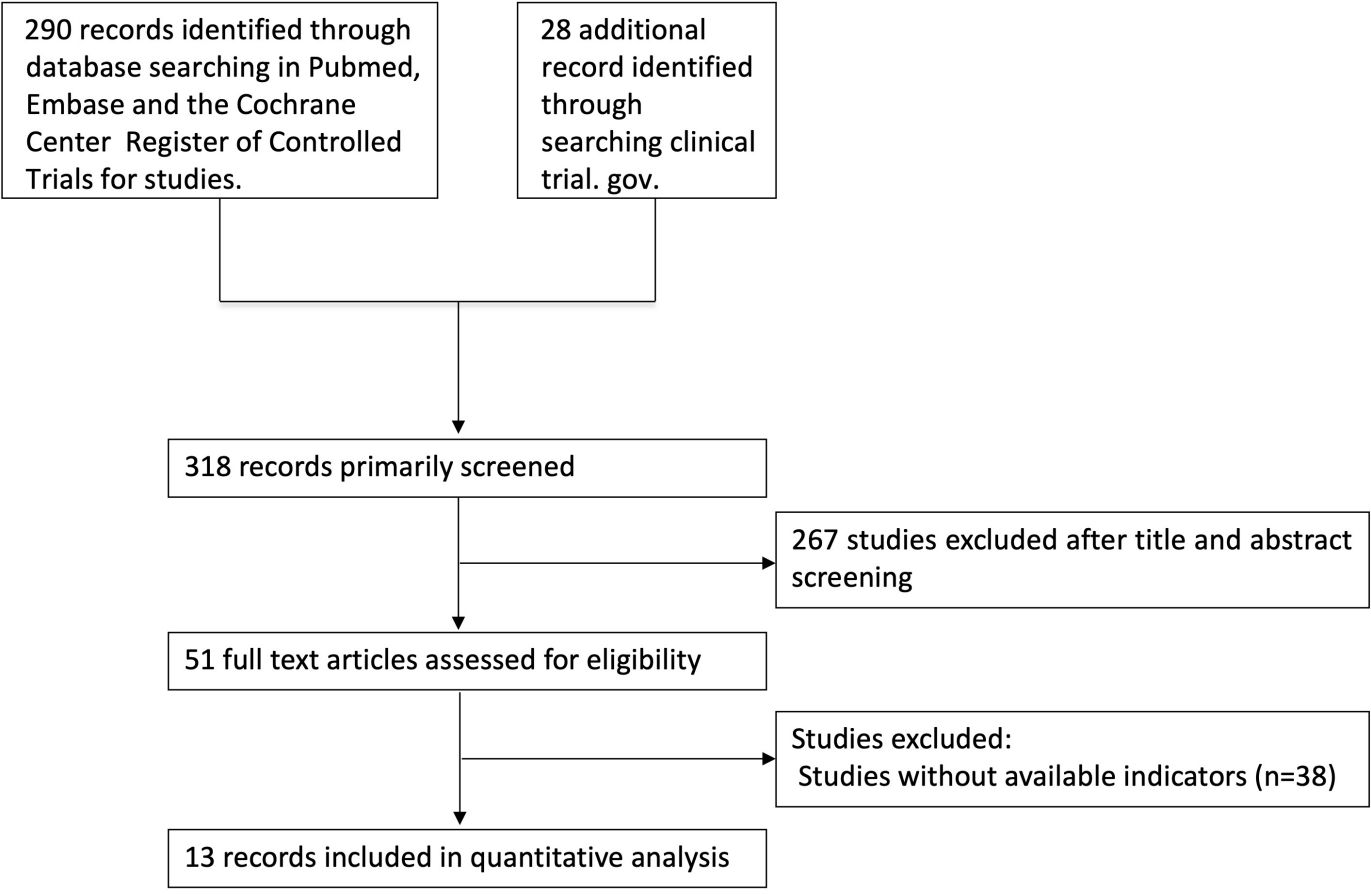
Flow diagram of the included studies.

In total, 13 placebo-controlled studies were included in this meta-analysis, among which are two RCTs with treatment of AZD 1656 in type 2 diabetes, three RCTs with treatment of dorzagliatin in type 2 diabetes, two RCTs with treatment of MK-0941 in type 2 diabetes, two studies with treatment of PB-201 in type 2 diabetes, two studies with treatment of PF-04991532 in type 2 diabetes, one study with treatment of TPP399 in type 2 diabetes, and one study with treatment of TPP399 in type 1 diabetes (including two separate parts) (**Figure 1**). The baseline characteristics and risk-of-bias evaluation for the included studies are summarized in **Table S2** and **Table S3**. The overall risk of bias was low. Funnel plots and Egger’s test showed even distributions (P = 0.416) (**Figure S1**).

#### Type 2 diabetes

##### Efficacy results

**Figure 2:**
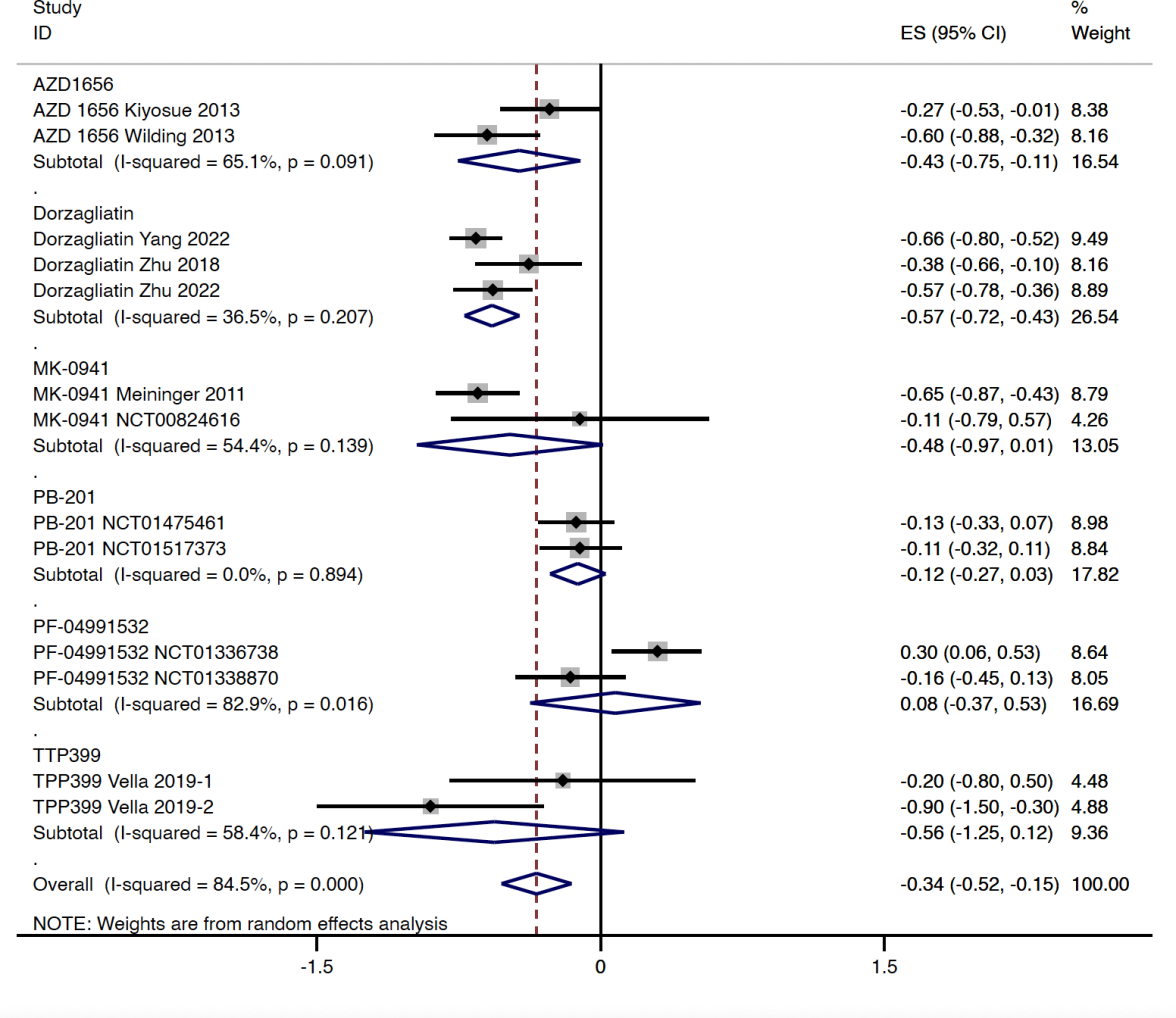
HbA1c change from baseline with GKA treatment versus placebo in type 2 diabetes. HbA1c, hemoglobin A1c; GKA, glucokinase activator; ES, effect size; CI, confidential interval.

In type 2 diabetes patients, compared with the placebo group, GKA treatment was associated with a greater reduction in the level of HbA1c (WMD = -0.339%, 95% CI -0.524 to -0.154%, P < 0.001; predictive interval -1.03 to 0.35; *I* ^2 ^= 84.5%). Greater reductions in HbA1c were observed in the AZD 1656 subgroup (WMD = -0.431%, 95% CI -0.754 to -0.107%, P = 0.009; *I* ^2 ^= 65.1%) and dorzagliatin subgroup (WMD = -0.575%, 95% CI -0.719 to -0.430%, P < 0.001; *I* ^2 ^= 36.5%), whereas no significant HbA1c change differences were observed in the MK-0941 subgroup (WMD = -0.480%, 95% CI -0.972 to 0.012%, P = 0.056; *I* ^2 ^= 54.4%), PB-201 subgroup (WMD = -0.121%, 95% CI -0.267 to 0.026%, P = 0.106; *I* ^2 ^= 0.0%), PF-04991532 subgroup (WMD = 0.078, 95% CI -0.372 to 0.529, P = 0.734, *I* ^2 ^= 82.9%), or TTP399 subgroup (WMD = -0.562, 95% CI -1.247 to 0.124, P = 0.108, *I* ^2 ^= 58.4%) (**Figure 2**). As for duration of follow-up, greater HbA1c reductions were observed in RCTs with longer follow-up (stratified by 12 and 24 weeks, respectively) (**Figures S2A, B**). In terms of selectivity, dual-acting GKAs (WMD = -0.410%, 95% CI -0.578 to -0.242%, P < 0.001; *I* ^2 ^= 78.2%) and hepatoselective GKAs (WMD = -0.183%, 95% CI -0.638 to 0.271%, P = 0.429; *I* ^2 = ^81.7%) showed a difference in the effects on HbA1c (**Figure S2C**).

**Figure 3:**
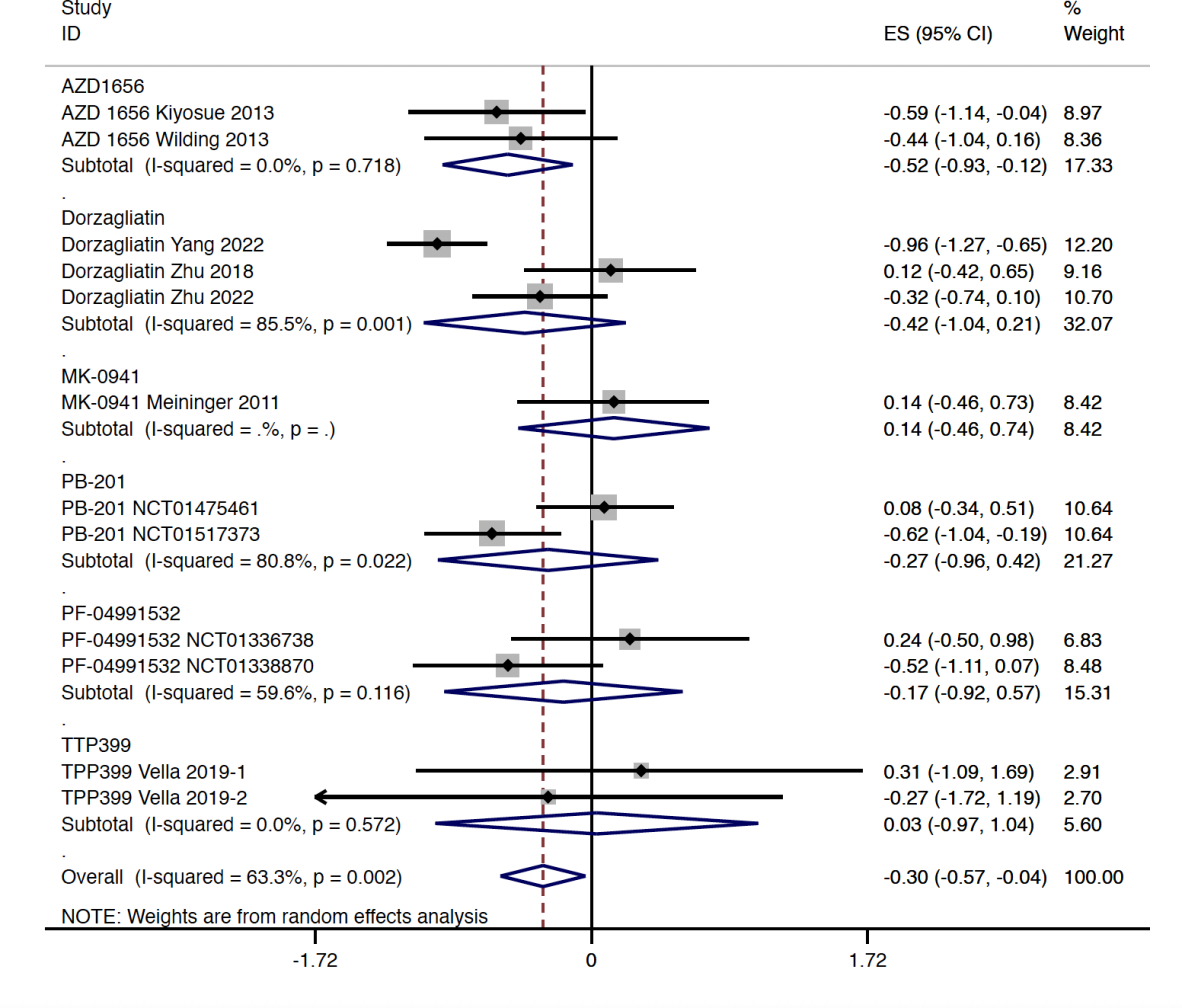
FPG change from baseline with GKA treatment versus placebo in type 2 diabetes. FPG, fasting plasma glucose; GKA, glucokinase activator; ES, effect size; CI, confidential interval.

Compared with the placebo group, the level of FPG was decreased slightly in patients with GKA treatment (WMD = -0.302 mmol/L, 95% CI -0.566 to -0.037 mmol/L, P = 0.025; predictive interval -1.14 to 0.54; *I* ^2 ^= 63.3%), which was mainly driven by the AZD 1656 subgroup (WMD = -0.522 mmol/L, 95% CI -0.927 to -0.116 mmol/L, P = 0.012; *I* ^2 = ^0.0%) (**Figure 3**). The study-duration analysis indicated that greater reductions in FPG were observed in RCTs with follow-up longer than 12 weeks (WMD = -0.422 mmol/L, 95% CI -0.775 to -0.070 mmol/L, P = 0.019; *I* ^2 ^= 59.4%) (**Figures S3A, B**). Similarly with the results of HbA1c, greater changes in FPG were found in RCTs with dual-acting GKAs (WMD = -0.346 mmol/L, 95% CI -0.660 to -0.031 mmol/L, P = 0.031; *I* ^2 ^= 72.8%) (**Figure S3C**).

Four studies reported the change of PPG from baseline, among which are three RCTs with treatment of dorzagliatin and one RCT with treatment of MK-0941. Compared with the placebo group, GKA treatment was associated with greater reduction in the level of PPG (WMD = -2.360 mmol/L, 95% CI -2.715 to -2.006 mmol/L, P < 0.001; predictive interval -3.14 to -1.58; *I* ^2 ^= 0.0%). The sensitivity analysis by drug type and study duration showed consistent results (**Figures S4A–C**).

When compared with the placebo group, GKA treatment was associated with a greater decrease in FINS (WMD = -0.964 μIU/ml, 95% CI -1.317 to -0.611 μIU/ml, P < 0.001; predictive interval -1.58 to -0.34; *I* ^2 ^= 9.0%) (**Table S4**). In addition, compared with the placebo group, a greater decrease in HOMA-IR (WMD = -0.077, 95% CI -0.133 to -0.022, P = 0.006; predictive interval -0.44 to 0.28; *I* ^2 ^= 0.0%) and a greater increase in HOMA-β (WMD = 2.681, 95% CI 1.141 to 4.221, P = 0.001; *I* ^2 ^= 0.0%) were revealed in the GKA (all with dorzagliatin treatment) group (**Table S4**).

##### Safety results

**Figure 4:**
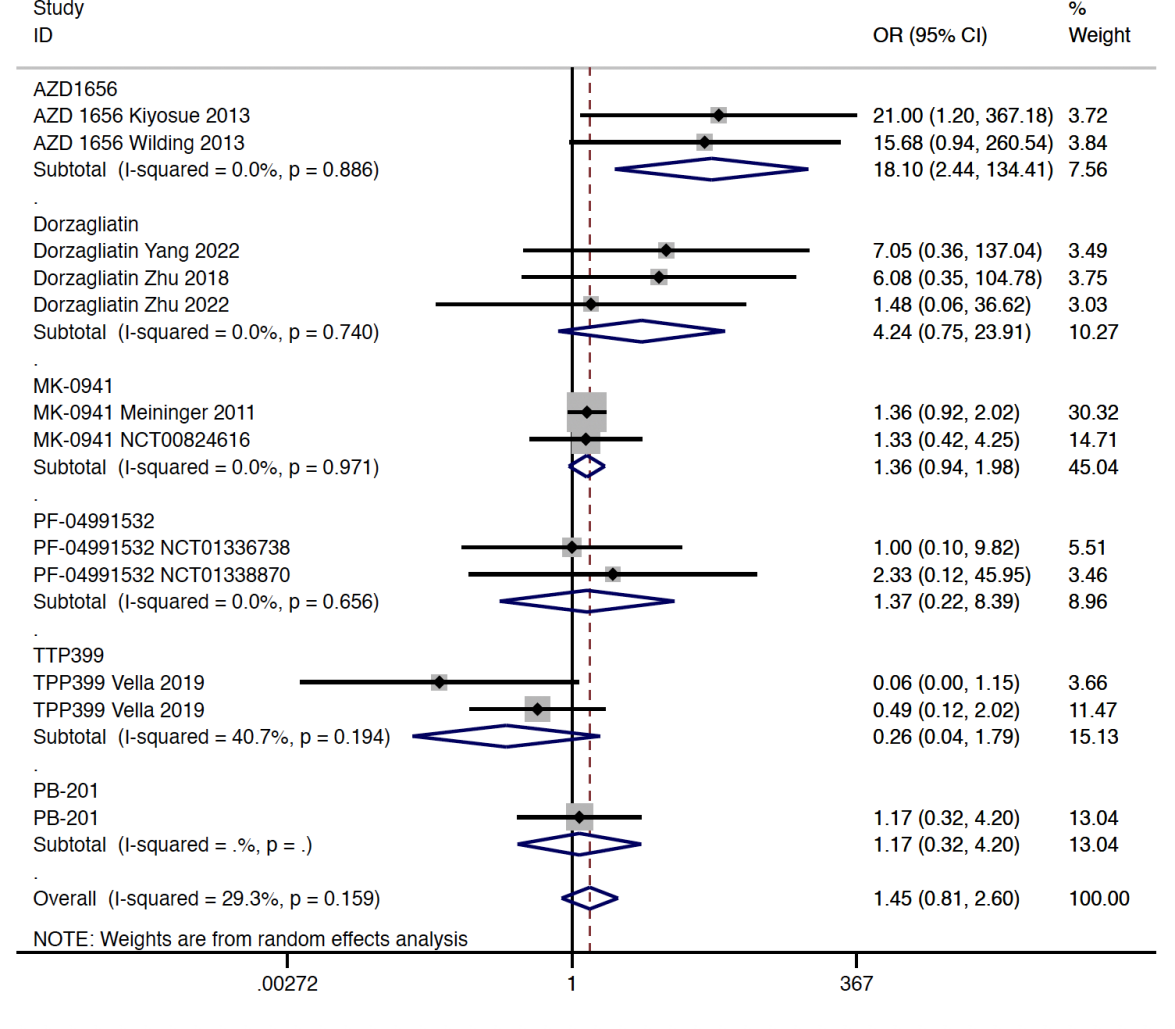
Incidence of hypoglycemia with GKA treatment versus placebo in type 2 diabetes. GKA, glucokinase activator; OR, odd ratio; CI, confidential interval.

In type 2 diabetes, results from this meta-analysis indicated that GKA treatment showed a similar risk of hypoglycemia when compared with the placebo treatment (OR = 1.448, 95% CI 0.808 to 2.596, P = 0.214; predictive interval 0.39 to 5.32; *I* ^2 ^= 29.3%). Among the investigated agents, the AZD 1656 subgroup (OR = 18.100, 95% CI 2.438 to 134.406, P = 0.005; *I* ^2 ^= 0.0%) was found to be associated with increased risk of hypoglycemia (**Figure 4**). Compared with the placebo group, treatment of dorzagliatin (OR = 4.241, 95% CI 0.752 to 23.915, P = 0.102; *I* ^2 ^= 0.0%), MK-0941 (OR = 1.361, 95% CI 0.938 to 1.976, P = 0.105; *I* ^2 ^= 0.0%), TPP399 (OR = 0.256, 95% CI 0.037 to 1.795, P = 0.170; *I* ^2 ^= 40.7%), PB-201 (OR = 1.165, 95% CI 0.323 to 4.199, P = 0.815), and PF-04991532 (OR = 1.368, 95% CI 0.223 to 8.387, P = 0.735; *I* ^2 ^= 0.0%) showed a similar risk of hypoglycemia in type 2 diabetes (**Figure 4**). The sensitivity analyses by study duration showed consistent results (**Figures S5A, B**). As for selectivity, increased risk of hypoglycemia was only observed in the dual-acting GKA group (OR = 1.884, 95% CI 1.023 to 3.469, P = 0.042; *I* ^2 ^= 24.0%) (**Figure S5C**).

**Figure 5:**
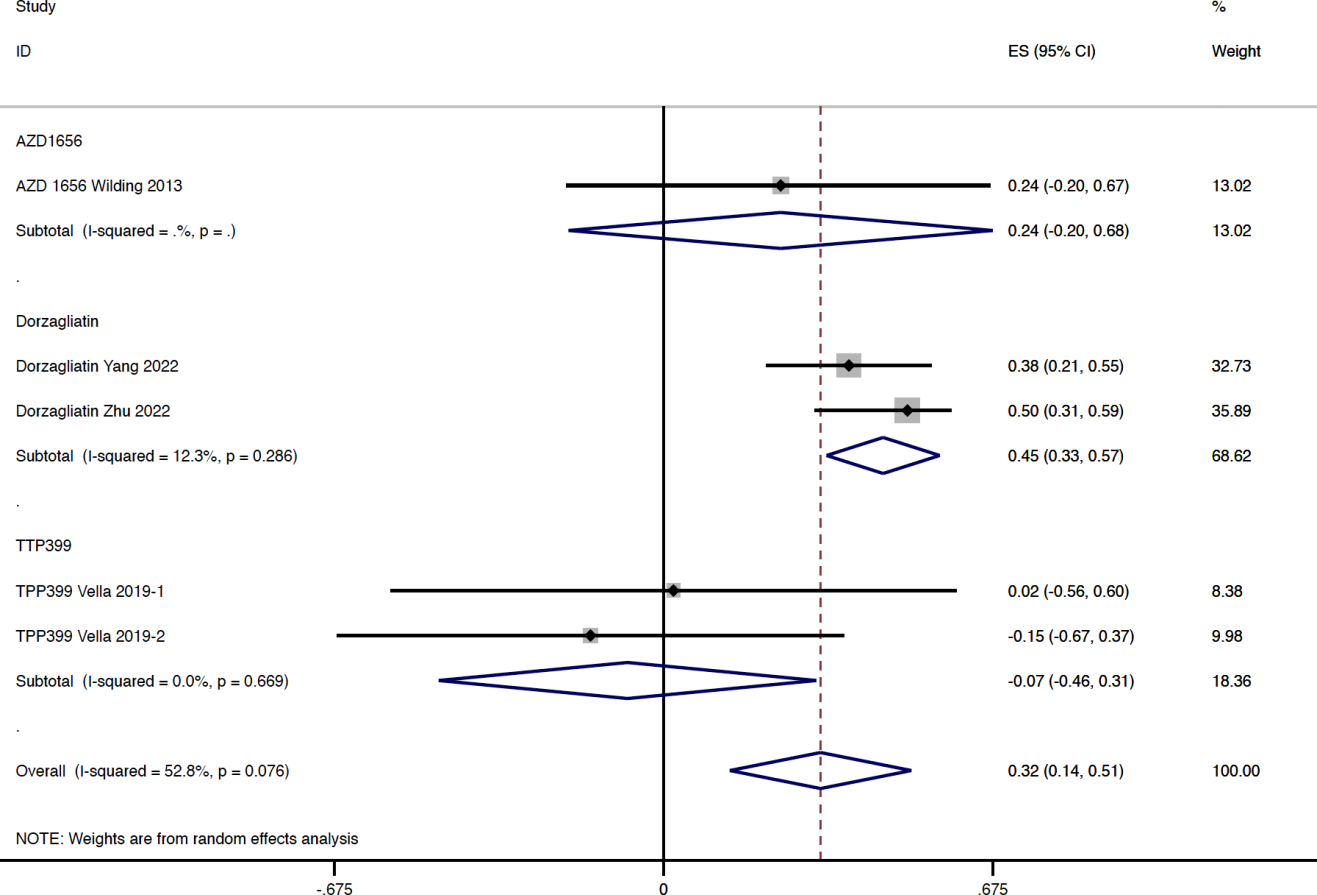
TG change from baseline with GKA treatment versus placebo in type 2 diabetes. TG, triglyceride; GKA, glucokinase activator; ES, effect size; CI, confidential interval.

In total, four studies reported available data regarding the change of blood lipid and uric acid concentration in type 2 diabetes. Compared with the placebo group, GKA treatment was associated with a greater elevation in TG concentration (WMD = 0.322 mmol/L, 95% CI 0.136 to 0.508 mmol/L, P = 0.001; predictive interval -0.22 to 0.86; *I* ^2 ^= 52.8%). The drug-type analysis indicated that the significant increase in TG level was observed in the dorzagliatin subgroup (WMD = 0.450 mmol/L, 95% CI 0.334 to 0.566 mmol/L, P < 0.001; *I* ^2 ^= 12.3%), but not in the AZD 1656 subgroup (WMD = 0.240 mmol/L, 95% CI -0.195 to 0.675 mmol/L, P = 0.280; *I* ^2 ^= 0.0%) or TTP399 subgroup (WMD = -0.074 mmol/L, 95% CI -0.461 to 0.313 mmol/L, P = 0.707; *I* ^2 ^= 0.0%) (**Figure 5**). In addition, the change of TG concentration from baseline was significantly greater in RCTs with dual-acting GKAs (WMD = 0.439 mmol/L, 95% CI 0.334 to 0.544 mmol/L, P < 0.001; *I* ^2 ^= 0.0%), when compared with the placebo group (**Figure S6**). In terms of other lipid indicators, one RCT with treatment of TPP399 and two RCTs with treatment of dorzagliatin reported available data in type 2 diabetes. Compared with the placebo group, GKA treatment was associated with a slight elevation of TCHO level (WMD = 0.136 mmol/L, 95% CI 0.057 to 0.214 mmol/L, P = 0.001; *I* ^2 ^= 0.0%). Increased SUA levels (WMD = 29.07 μmol/L, 95% CI 18.11 to 40.03 μmol/L, P < 0.001; *I* ^2 ^= 52.7%) were observed in the GKA group (all were RCTs with dorzagliatin treatment) compared with placebo users (**Table S5**).

In terms of liver safety, significant elevations in ALT (WMD = 4.146 U/L, 95% CI 2.339 to 5.988 U/L, P < 0.001; *I* ^2 ^= 38.0%) and AST (WMD = 4.26 U/L, 95% CI 3.574 to 5.477 U/L, P < 0.001; *I* ^2 ^= 0.0%) were observed after treatment with GKA as compared with placebo (**Figures S7**,**S8**). The change in LDL-C, HDL-C, and eGFR from baseline showed no significant difference between the GKA group and placebo group (**Table S5**).

#### Type 1 diabetes

##### Efficacy results

In type 1 diabetes, HbA1c was evaluated in one RCT with two separate parts, whereas the aggregated result of HbA1c change showed no significant difference between the TPP399 group and placebo group (WMD = -0.366%, 95% CI -0.800 to -0.068%, P = 0.098; *I* ^2 ^= 60%) (**Figure S9**).

##### Safety results

In type 1 diabetes, TG concentration change from baseline showed no significant difference between the TTP399 treatment and placebo groups (WMD = 0.036 mmol/L, 95% CI -0.355 to 0.427 mmol/L, P = 0.857; *I* ^2 ^= 75.8%). Results of TCHO change, LDL-C change, or HDL-C change from baseline showed no significant difference between the TPP399 group and placebo group (**Table S6**).

#### Associated factors with the GKA efficacy and safety

In type 2 diabetes, meta-regression analysis results showed that covariates such as age, gender, duration of diabetes, BMI, baseline HbA1c, FPG change, TG change from baseline, and FPG change from baseline was associated with the change of HbA1c in the GKA group when compared with the placebo group (**Table S7**). The above covariates and change of HbA1c were not associated with the risk of hypoglycemia (**Table S8**) or change of TG concentration (**Table S9**) in the GKA group when compared with the placebo group.

## Discussion

This is the first meta-analysis systematically evaluating efficacy and safety of GKA treatment. In the current study, we found that treatment with GKA showed greater reduction in HbA1c, FPG, and PPG when compared with the placebo group. The sensitivity analysis demonstrated a difference when stratified by drug type, selectivity, and study duration. Regarding the safety profiles, risk of hypoglycemia showed no significant difference between the GKA group and placebo group, whereas a significant elevation in TG concentration was observed in GKA users. In addition, levels of ALT, AST, and SUA appeared to be associated with treatment with GKA in some studies, especially in studies with dorzagliatin.

GK is the enzyme catalyzing glucose phosphorylation, which expressed primarily in beta cells and hepatocytes. In the pancreas, GK establishes the threshold for GSIS, whereas in the liver, it regulates glucose uptake and controls glycogen synthesis (2, 3). However, the research and development process of GKA is challenging; only a minority have reached the clinical trial stage. According to the pooled results in this meta-analysis, GKA treatment demonstrated an improved effect on glycemic control in type 2 diabetes, with a placebo-adjusted HbA1c reduction ranging from -0.12% to -0.57%, except for PF-04991532. Among these GKAs, dorzagliatin and TTP399 800 mg once daily appeared to be superior in HbA1c improvement. The placebo-adjusted FPG decreased slightly ranged from -0.17 to -0.52 mmol/L, whereas the placebo-adjusted PPG reduction ranged from -1.88 to -2.47 mmol/L. It seems that the improvement observed in HbA1c was primarily derived from the change in PPG. The efficacy results indicated that GKA treatment only showed a slight improvement in glycemia control. It is of note that results from the previous UK Prospective Diabetes Study (UKPDS) indicated that the minimum clinically important differences (MCIDs) for HbA1c in type 2 diabetes was 1%, which bring on improvement of clinical complications (15). Therefore, does the slight improvement in glycemia control observed in GKA treatment bring benefits for type 2 diabetes patients? Large cardiovascular outcomes trials (CVOTs) are needed to answer this question. In addition, head-to-head studies directly comparing the efficacy of GKAs and other hypoglycemic agents are expected in the future studies.

In this meta-analysis, we observed a significant decrease in FINS level with treatment of GKAs when compared with placebo. Among the included studies, the concentration of FINS declined significantly with treatment of AZD1656 and PB-201, whereas no significant difference in FINS was found in treatment of dorzagliatin or TPP399 when compared with placebo. The pooled analysis from dorzagliatin showed favorable effects in both HOMA-IR and HOMA-β. Unfortunately, results regarding HOMA-IR and HOMA-β were not available for other GKA agents in the included studies. Additionally, a previous study indicated that activation of GK was associated with increased level of glucagon-like peptide-1 (GLP-1) (5), whereas few included studies provide relevant results. More studies with thorough evaluation of islet cell function, insulin resistance, and effects on incretins would provide comprehensive insight into the mechanism of action of GKAs.

GKAs could be classified into full-acting activator and hepatoselective activator (5). The original intension for developing hepatoselective activator was to reduce the potential pancreatic GK overactivating-related adverse effects. Of note, significant improvement in HbA1c and FPG was not observed in hepatoselective GKAs from this meta-analysis, including TTP399 and PF-04991532. A lack of effectiveness was the prominent obstacle in the previous development process of PF-04991532. Meanwhile, in the two-dose arms of TTP399 conducted in type 2 diabetes, significant glycemic improvement was only observed in the high-dose group of 800 mg once daily when compared with placebo. Therefore, the overall effect size of hepatoselective GKAs on glycemic control was weakened by the PF-04991532 and TPP399 400 mg once daily groups. Whether the pancreas and liver dual-acting activator play a stronger effect on glycemic control than the hepatoselective activator is still unknown. The result from the TPP399 800 mg once daily arm, after all, demonstrated a significant effectiveness on glycemic control with a good safety profile during the 6-month treatment (16). More evidence is expected from clinical trials evaluating the efficacy and safety of hepatoselective activator.

Currently, one of the most concerned issues regarding GKAs was the attenuated efficacy over time, which was observed in previous studies (7, 9). The possible explanation for the observed lack of sustained efficacy for MK-0941 was that participants with long duration of diabetes were included, who had severe beta cell impairment. It was explained that GKA was unable to exert a pancreatic effect on this kind of patients. For the lack of sustained efficacy of AZD 1656, it was speculated that the glucolipotoxicity from chronic ongoing activation of the GK in the pancreatic beta cell led to a reduction in glucose responsive islets (17–19). According to our analysis, no attenuated efficacy over time was found in the changes of HbA1c. Studies with a longer duration of follow-up are further needed to fully elucidate this issue.

Increased risk of hypoglycemia, which was associated with the GK overstimulation and subsequent disruption of the GSIS threshold (5, 17, 18), is a pronounced adverse effect that hindered the development of GKAs. According to this meta-analysis, the overall risk of hypoglycemia was similar when compared with placebo, whereas the dual-activator subgroup showed an significant increased risk of hypoglycemia. Conversely, compared with placebo, the risk of hypoglycemia was similar with the treatment of hepatoselective activators TTP399 and PF-04991532. This finding was consistent with the mechanism proposed above exactly. Effectively balancing efficacy and risk of hypoglycemia remains to be an essential issue to be solved.

The occurrence of dyslipidemia was another concern regarding GKAs. The activation of GK was required for the initial step of the hepatic lipogenic pathway, and GK mRNA expression was associated with markers of *de novo* lipogenesis and liver triglyceride content in humans. Based on these physiological pathways, it had been speculated that the inappropriate activation of liver GK could result in elevations of TG concentrations (5, 19). For patients with diabetes who were already at higher risk of cardiovascular complication, dyslipidemia was an undesired adverse effect. More than that, hepatic steatosis, steatohepatitis, and ultimate cirrhosis induced by chronic elevation of TG should also be considered (19). According to this meta-analysis, the pooled TG level was demonstrated to be increased significantly in the dorzagliatin group when compared with placebo. Furthermore, estimation of laboratory indicators suggested an elevation in ALT, AST, and SUA in patients receiving dorzagliatin. There is a high likelihood that the elevation of ALT, AST, and SUA is the side effect of dorzagliatin as evidenced by the fact that when placebo-treated study subjects were switched to dorzagliatin treatment in the clinical trials, ALT, AST, and SUA all increased to the same level as that in dorzagliatin treatment groups (20). Were the unfavorable changes in these metabolic parameters transient or continuous? Based on the current limited evidence, the answer was not available. However, previous genome-wide association studies might provide some clues. These studies revealed that glucokinase regulator protein (GCKR) single-nucleotide polymorphisms (SNPs) were associated with the elevated level of SUA and the increased risk of gout (21–25). Further investigations are needed to elucidate the clinical implications of those unfavorable changes and the underlying mechanism.

In this meta-analysis, we also synthesized the two parts of SimpliciT1 Study performed in type 1 diabetes. In patients with type 1 diabetes, hepatic exposure to insulin was subnormal, resulting in less GK expression and subsequent compromised liver uptake and metabolism of glucose (26). Additionally, restoring GK expression in the liver improved glycemic control in streptozotocin-induced type 1 diabetic rats (27). In this context, the SimpliciT1 Study was conducted to determine the efficacy and safety of TTP399 in type 1 diabetes (28). The pooled HbA1c change from baseline was not statistically significant with TPP399 treatment when compared with placebo. However, participants assigned to TPP399 in both parts achieved better glycemic control with numeric reduction in insulin doses. Importantly, TPP399 treatment was found to be associated with a reduced risk of hypoglycemia and a reduced risk of ketosis. Given the above observations, TTP399 appeared to be a promising adjunct therapy to insulin in the treatment of type 1 diabetes and merited more extensive evaluations.

This meta-analysis also has some limitations. First, we combined data from trials that varied in study duration, interventions, and baseline characteristics. However, we used the random-effect model for statistical analysis and performed multiple sensitivity analyses to control the heterogeneity. Second, some included RCTs were dose-finding studies containing diverse doses. Nevertheless, as a class of agents under exploration and development, the optimal dose still could not be determined for some GKAs. Thus, the deviations in results brought by the non-optimal doses could not be avoided in the current meta-analysis. Third, the complete follow-up information could not be obtained from the published data in part of the included studies, leading to reporting bias to the pooled analysis. Fourth, the study duration of included studies was relatively short, weakening the power to examine the long-term efficacy and safety. Fifth, limited computable laboratory indicators hindered the in-depth evaluation of metabolic and safety issues.

In conclusion, according to the results of this meta-analysis, GKA treatment was associated with a better glycemic control. Regarding the safety profiles, risk of hypoglycemia showed no significant difference between the GKA group and placebo group, whereas a significant elevation in TG concentration was observed in GKA users. The efficacy and safety of GKA treatment varied with drug type and selectivity. More studies with a longer follow-up duration and larger sample size are still needed to provide solid evidence to evaluate the long-term efficacy and safety of GKAs.

## Data availability statement

The original contributions presented in the study are included in the article/**Supplementary Material**. Further inquiries can be directed to the corresponding authors.

## Author contributions

LJ and XC designed the study. WY and HW conducted the data collection. WY, XC, CL, and RJ conducted the statistical analysis. WY drafted the manuscript. All authors contributed to the article and approved the submitted version.
