# Supplementary material for: Evaluation of efficacy and safety of glucokinase activators—a systematic review and meta-analysis
Source: Front Endocrinol (Lausanne). 2023 May 8;14:1175198. doi: 10.3389/fendo.2023.1175198 (PMC10200948; doi:10.3389/fendo.2023.1175198)
Supplement: Supplementary file 1 [file DataSheet_1.docx]

Supplementary Figures:

Figure S1. Funnel plot assessing publication bias

Figure S2A. HbA1c changes from baseline with GKAs treatment versus placebo stratified by the duration of follow-up (12 weeks) in T2DM

Figure S2B. HbA1c changes from baseline with GKAs treatment versus placebo stratified by the duration of follow-up (24 weeks) in T2DM

Figure S2C. HbA1c changes from baseline with GKAs treatment versus placebo stratified by selectivity in T2DM

Figure S3A. FPG changes from baseline with GKAs treatment versus placebo stratified by the duration of follow-up (12 weeks) in T2DM

Figure S3B. FPG changes from baseline with GKAs treatment versus placebo stratified by the duration of follow-up (24 weeks) in T2DM

Figure S3C. FPG changes from baseline with GKAs treatment versus placebo stratified by selectivity in T2DM

Figure S4A. PPG changes from baseline with GKAs treatment versus placebo stratified by drug type in T2DM

Figure S4B. PPG changes from baseline with GKAs treatment versus placebo stratified by the duration of follow-up (12 weeks) in T2DM

Figure S4C. PPG changes from baseline with GKAs treatment versus placebo stratified by the duration of follow-up (24 weeks) in T2DM

Figure S5A. Risk of hypoglycemia with GKAs treatment versus placebo stratified by the duration of follow-up (12 weeks) in T2DM

Figure S5B. Risk of hypoglycemia with GKAs treatment versus placebo stratified by the duration of follow-up (24 weeks) in T2DM

Figure S5C. Risk of hypoglycemia with GKAs treatment versus placebo stratified by selectivity in T2DM

Figure S6. TG changes from baseline with GKAs treatment versus placebo stratified by selectivity in T2DM

Figure S7. ALT changes from baseline with GKAs treatment versus placebo

Figure S8. AST changes from baseline with GKAs treatment versus placebo

Figure S9. HbA1c changes from baseline with GKAs treatment versus placebo in T1DM

Supplementary Tables:

Table S1. Search strategy

Table S2. Baseline characteristics of included studies

Table S3. Risk of bias of included studies

Table S4. FINS, HOMA-β and HOMA-IR change from baseline with GKAs treatment versus placebo

Table S5. Metabolic and safety indicators with GKAs treatment versus placebo.

Table S6. Metabolic and safety indicators with GKAs treatment versus placebo in type 1 diabetes.

Table S7. Meta-regression analyses for the associated factors with HbA1c change

Table S8. Meta-regression analyses for the associated factors with TG change

Table S9. Meta-regression analyses for the associated factors with risk of hypoglycemia

Figure S1. Funnel plot assessing publication bias


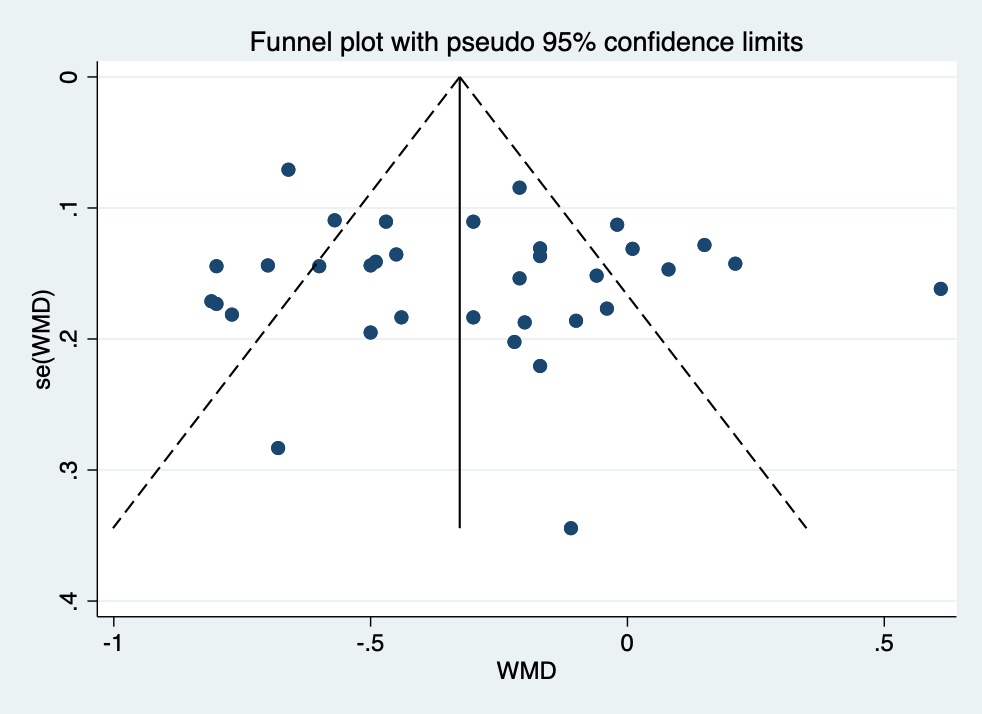


Figure S2A. HbA1c changes from baseline with GKAs treatment versus placebo stratified by the duration of follow-up (12 weeks) in T2DM

Figure S2B. HbA1c changes from baseline with GKAs treatment versus placebo stratified by the duration of follow-up (24 weeks) in T2DM

Figure S2C. HbA1c changes from baseline with GKAs treatment versus placebo stratified by selectivity in T2DM

Figure S3A. FBG changes from baseline with GKAs treatment versus placebo stratified by the duration of follow-up (12 weeks) in T2DM

Figure S3B. FBG changes from baseline with GKAs treatment versus placebo stratified by the duration of follow-up (24 weeks) in T2DM

Figure S3C. FBG changes from baseline with GKAs treatment versus placebo stratified by selectivity in T2DM

Figure S4A. PPG changes from baseline with GKAs treatment versus placebo stratified by drug type in T2DM

Figure S4B. PPG changes from baseline with GKAs treatment versus placebo stratified by the duration of follow-up (12 weeks) in T2DM

Figure S4C. PPG changes from baseline with GKAs treatment versus placebo stratified by the duration of follow-up (24 weeks) in T2DM

Figure S5A. Risk of hypoglycemia with GKAs treatment versus placebo stratified by the duration of follow-up (12 weeks) in T2DM

Figure S5B. Risk of hypoglycemia with GKAs treatment versus placebo stratified by the duration of follow-up (24 weeks) in T2DM

Figure S5C. Risk of hypoglycemia with GKAs treatment versus placebo stratified by selectivity in T2DM

Figure S6. TG changes from baseline with GKAs treatment versus placebo stratified by selectivity in T2DM

Figure S7. ALT changes from baseline with GKAs treatment versus placebo

Figure S8. AST changes from baseline with GKAs treatment versus placebo

Figure S9. HbA1c changes from baseline with GKAs treatment versus placebo in T1DM


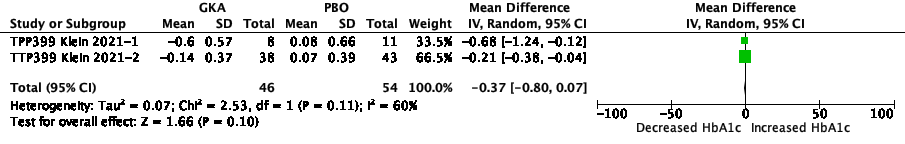


Table S1. Search strategy

| Databases | Search items |
| --- | --- |
| Pubmed  (n=99) | (("glucokinase"[All Fields] AND "activators"[All Fields]）OR "glucokinase activators"[All Fields] OR "glucokinase activator"[All Fields] OR ("GK"[All Fields] AND "activator*"[All Fields]) OR ("GCK"[All Fields] AND "activator*"[All Fields]) OR “glucokinase”[All Fields] OR “GCK”[All Fields] OR “Dorzagliatin”[All Fields] OR “HMS5552” [All Fields] OR “Piragliatin” [All Fields] OR “RO4389620” [All Fields] OR “AMG 151” [All Fields] OR “ARRY-403” [All Fields] OR “AZD1656” [All Fields] OR “AZD6370” [All Fields] OR “TMG-123” [All Fields] OR “MK-0941” [All Fields] OR “TTP-399” [All Fields] OR “GKI-399” [All Fields] OR “SY004” [All Fields] OR “GKM001” [All Fields] OR “globalagliatin” [All Fields] OR “LY2608204” [All Fields] OR “PB-201” [All Fields] OR “PF-04937319” [All Fields] OR “PF-04991532” [All Fields]） AND ("randomized controlled trial"[Publication Type] OR "controlled clinical trial"[Publication Type] OR "randomized"[Title/Abstract] OR "randomly"[Title/Abstract] OR "random*"[Title/Abstract]) AND (humans[Filter]) |
| Embase  (n=122) | #1 ‘glucokinase activator’ OR ‘glucokinase activator*’ OR ‘glucokinase’ OR ‘gka’ OR ‘gck’  #2 Dorzagliatin OR HMS5552 OR Piragliatin OR RO4389620 OR ‘AMG 151’ OR ARRY-403 OR ‘AZD1656’ OR AZD6370 OR TMG-123 OR MK-0941 OR TTP-399 OR SY004 OR GKM001 OR globalagliatin OR LY2608204 OR PB-201 OR PF-04937319 OR PF-04991532 OR GKI-399  #3 #1 OR #2  #4 ‘randomi*ed controlled trial’ OR ‘randomi*ed trial’  #5 #3 AND #4 |
| CENTRAL  (n=69) | (“glucokinase activator” or “glucokinase activator*” or “GKA” or “GKA*” or “GK” or “GCK”) or (“Dorzagliatin” or “HMS5552” or “Piragliatin” or “RO4389620” or “AMG 151” or “ARRY-403” or “AZD1656” or “AZD6370” or “TMG-123” or “MK-0941” or “TTP-399” or “SY004” or “GKM001” or “globalagliatin” or “LY2608204” or “PB-201” or “PF-04937319” or “PF-04991532” or “GKI-399”) in Title Abstract Keyword AND ("randomi?ed trial" OR "randomi?ed" OR "random allocation" OR random* OR "controlled trial" OR "randomized controlled trial" ) in Title Abstract Keyword - (Word variations have been searched) |

Table S2. Baseline characteristics of included studies

| Author, year | Duration of follow-up | Treatment group | Number of participants | Age (years) | Male (%) | BMI (kg/m^2^) | Baseline Weight (kg) | Baseline HbA1c （%） |
| --- | --- | --- | --- | --- | --- | --- | --- | --- |
| AZD1656 |  |  |  |  |  |  |  |  |
| Kiyosue 2013 [1] | 4 months | AZD1656 10-80mg | 56 | 55±10 | 85.7 | 26.5±4.4 | / | 8.9±1 |
|  |  | AZD1656 20-140mg | 58 | 55±9 | 86.2 | 25.8±4.3 | / | 8.6±0.9 |
|  |  | AZD1656 40-200mg | 55 | 55±9 | 87.3 | 25.3±3.3 | / | 8.7±1 |
|  |  | PBO | 55 | 57±9 | 83.6 | 26.1±4 | / | 8.4±0.7 |
| Wilding 2013 [2] | 4 months | AZD1656 20mg+MET | 40 | 57.4±9.2 | 47.5 | 32.4±4.9 | / | 8.2±0.9 |
|  |  | AZD1656 40mg+MET | 52 | 54.4±9.1 | 42.3 | 31.2±4.8 | / | 8.4±0.7 |
|  |  | AZD1656 10-140mg+MET | 91 | 57.1±9.3 | 50.5 | 30.5±4.5 | / | 8.4±0.9 |
|  |  | AZD1656 20-200mg+MET | 93 | 57.1±8.5 | 50.5 | 30.8±4.5 | / | 8.4±0.8 |
|  |  | PBO+MET | 88 | 56.9±9.6 | 51.1 | 30.6±4.9 | / | 8.3±0.9 |
| Dorzagliatin |  |  |  |  |  |  |  |  |
| Yang 2022 [3] | 24 weeks | Dorzagliatin 75mg bid+MET | 382 | 54.6±10 | 64 | 25.7±3 | 70.8±11.4 | 8.3±0.6 |
|  |  | PBO+MET | 385 | 54.4±9.2 | 60 | 26.1±3.2 | 71.5±12.2 | 8.3±0.6 |
| Zhu 2018 [4] | 12 weeks | Dorzagliatin 75mg qd | 53 | 57.6±9.2 | 51 | 24.72±2.87 | / | 8.44±0.8 |
|  |  | Dorzagliatin 100mg qd | 50 | 56.7±7.7 | 56 | 25.01±2.94 | / | 8.27±0.64 |
|  |  | Dorzagliatin 50mg bid | 50 | 54.9±8.1 | 69 | 24.69±2.31 | / | 8.33±0.65 |
|  |  | Dorzagliatin 75mg bid | 49 | 55.4±7.7 | 63 | 25.32±2.54 | / | 8.46±0.67 |
|  |  | PBO | 53 | 54.7±8.5 | 58 | 25.19±2.6、 | / | 8.39±0.78 |
| Zhu 2022 [5] | 24 weeks | Dorzagliatin 75mg bid | 310 | 53.2±9.6 | 65 | 25.6±2.9 | 69.8±10.8 | 8.3±0.7 |
|  |  | PBO | 153 | 53.5±10 | 66 | 25.3±2.7 | 68.3±9.8 | 8.4±0.7 |
|  |  | PBO | 75 | 52.7±10.3 | 41.3 | 32.3±5.5 | 88.8±19.0 | 7.84±0.87 |
| MK-0941 |  |  |  |  |  |  |  |  |
| Meininger 2011 [6] | 54weeks | MK0941 10mg+INS | 119 | 56.2±8.4 | 44 | 31.2±5.1 | / | 9.1±1.1 |
|  |  | MK0941 20mg+INS | 117 | 55.7±8.4 | 51 | 30.4±5.5 | / | 8.9±0.9 |
|  |  | MK0941 30mg+INS | 117 | 56.4±8.1 | 54 | 30.6±5.4 | / | 9.0±0.9 |
|  |  | MK0941 40mg+INS | 119 | 56.1±8.6 | 50 | 30.9±5.7 | / | 9.0±1.1 |
|  |  | PBO+INS | 115 | 56.4±9.2 | 50 | 30.8±5.3 | / | 9±0.9 |
| NCT00824616 [7] | 20weeks | MK-0941 5 or 10mg tid | 34 | 54.1±10.2 | 52.9 | / | / | / |
|  |  | PBO | 34 | 55.5±8.8 | 55.9 | / | / | / |
| PF-04937319 |  |  |  |  |  |  |  |  |
| NCT01475461 [8] | 12 weeks | PF-04937319 3mg | 57 | 48±5.3 | 49 | / | 88±24 | 8.0±1.1 |
|  |  | PF-04937319 20mg | 54 | 48±5.9 | 59 | / | 87±18 | 7.9±1.1 |
|  |  | PF-04937319 50mg | 56 | 47±7.3 | 59 | / | 88±20 | 8.2±1 |
|  |  | PF-04937319 100mg | 56 | 48±6.3 | 54 | / | 91±21 | 8.3±1.1 |
|  |  | PBO | 57 | 48±6.3 | 63 | / | 87±19 | 8.1±1.1 |
| NCT01517373 [9] | 12 weeks | PF-04937319 10mg+MET | 60 | 55±8.0 | 57 | / | 90±20 | 8.0±0.9 |
|  |  | PF-04937319 50mg+MET | 61 | 56±9.6 | 61 | / | 90±21 | 8.0±1.0 |
|  |  | PF-04937319 100mg+MET | 61 | 56±9.8 | 48 | / | 88±19 | 7.9±1.0 |
|  |  | PBO+MET | 61 | 55±8 | 56 | / | 90±22 | 7.9±1 |
| PF-04991532 |  |  |  |  |  |  |  |  |
| NCT01336738 [10] | 12 weeks | PF-04991532 150mg | 52 | 55.3±9.9 | 69.2 | / | / | 8.34±0.906 |
|  |  | PF-04991532 450mg | 54 | 55.1±9.3 | 55.6 | / | / | 8.19±0.947 |
|  |  | PF-04991532 750mg | 53 | 55.5±7.3 | 67.9 | / | / | 7.96±1.03 |
|  |  | PBO | 53 | 55.6±8.5 | 73.6 | / | / | 8.55±1.351 |
| NCT01338870 [11] | 12 weeks | PF-04991532 5mg bid | 49 | 59.2±6.8 | 38.8 | 25.4±3.54 | 69.7±13.7 | 7.9±0.939 |
|  |  | PF-04991532 75mg bid | 50 | 56.1±8.6 | 58 | 24.8±3.56 | 65.5±12.2 | 7.86±0.998 |
|  |  | PF-04991532 150mg bid | 50 | 57.2±8.7 | 60 | 24.5±3.21 | 66.3±12.4 | 7.93±1.018 |
|  |  | PF-04991532 300mg bid | 52 | 56±7.7 | 42.3 | 25.2±4.26 | 68.3±13.4 | 8.01±1.064 |
|  |  | PBO | 50 | 55.7±8.8 | 68 | / | / | 8.11±1.255 |
| TTP399 |  |  |  |  |  |  |  |  |
| Vella 2019 [12] | 6 months | TTP399 400mg | 50 | 52.86±10.7 | 46 | 33.3±5.4 | 94.1±19.4 | 8.45±0.75 |
|  |  | TTP399 800mg | 42 | 576±8.5 | 52.4 | 32.1±5.8 | 92.3±24.2 | 8.34±0.81 |
|  |  | PBO | 48 | 55.7±11.7 | 59.2 | 32.4±5.6 | 91.6±19.8 | 8.41±0.78 |
| Klein 2021 [13] | 12 weeks | TTP399 800mg | 8 | 38±15 | 37 | 28.4±3.3 | 28.4±3.3 | 7.2±0.4 |
|  |  | TTP399 800mg | 38 | 43±15 | 63 | 28.4±3.3 | 27.6±4 | 7.6±0.6 |
|  |  | PBO | 11 | 47±10 | 27 | 29±4.1 | 29±4.1 | 7.4±0.4 |
|  |  | PBO | 43 | 42±13 | 44 | 29±4.1 | 28.3±3.8 | 7.5±0.6 |

Abbreviations: MET: metformin; INS: insulin; SU: sulphonylurea; GI: glycemic index; PBO: placebo; BMI: body mass index; HbA1c: hemoglobin A1c; QD: once daily; BID: twice daily; TID: three times daily.

References:

1. Kiyosue A, Hayashi N, Komori H, Leonsson-Zachrisson M, Johnsson E. Dose-ranging study with the glucokinase activator AZD1656 as monotherapy in Japanese patients with type 2 diabetes mellitus. *Diabetes Obes Metab.* 2013;**15**(10):923–930. https://doi.org/10.1111/dom.12100.
2. Wilding JP, Leonsson-Zachrisson M, Wessman C, Johnsson E. Dose-ranging study with the glucokinase activator AZD1656 in patients with type 2 diabetes mellitus on metformin. *Diabetes Obes Metab*. 2013;**15**(8):750–759. https://doi.org/10.1111/dom.12088.
3. Yang W, Zhu D, Gan S, et al. Dorzagliatin add-on therapy to metformin in patients with type 2 diabetes: a randomized, double-blind, placebo-controlled phase 3 trial. *Nat Med.* 2022;**28**(5):974-981. https://doi.org/10.1038/s41591-022-01803-5.
4. Zhu D, Gan S, Liu Y, et al. Dorzagliatin monotherapy in Chinese patients with type 2 diabetes: a dose-ranging, randomised, double-blind, placebo-controlled, phase 2 study. *Lancet Diabetes Endocrinol.* 2018;**6**(8):627-636. https://doi: 10.1016/S2213-8587(18)30105-0.
5. Zhu D, Li X, Ma J, et al. Dorzagliatin in drug-naïve patients with type 2 diabetes: a randomized, double-blind, placebo-controlled phase 3 trial. *Nat Med.* 2022;**28**(5):965-973. https://doi.org/10.1038/s41591-022-01802-6.
6. Meininger GE, Scott R, Alba M, et al. Effects of MK-0941, a novel glucokinase activator, on glycemic control in insulin-treated patients with type 2 diabetes. *Diabetes Care.* 2011;**34**(12):2560–2566. https://doi.org/10.2337/dc11-1200.
7. A study to test MK-0941 in adults with type 2 diabetes mellitus with inadequate glycemic control on insulin (MK-0941-018). <https://clinicaltrials.gov/ct2/show/NCT00824616?term=MK-0941-018&draw=2&rank=1/;2015> [access September 02, 2022].
8. Phase 2 Study to evaluate safety and efficacy of investigational drug - PF04937319 in patients with type 2 diabetes. [https://clinicaltrials.gov/ct2/show/NCT01475461?term=PF04937319&draw=2&rank=1](https://clinicaltrials.gov/ct2/show/NCT01475461?term=NCT01475461&draw=2&rank=1)/;2017 [access September 02, 2022].
9. Study to understand efficacy and safety of investigational agent (PF-04937319) compared to approved agent (glimepiride) in patients with diabetes on metformin. <https://clinicaltrials.gov/ct2/show/NCT01517373?term=PF-04937319&draw=2&rank=1/;2017> [access September 05, 2022].
10. Study of safety and efficacy of PF-04991532 in subjects with type 2 diabetes mellitus. [https://clinicaltrials.gov/ct2/show/NCT01336738?term=PF-04991532&draw=2&rank=1](https://clinicaltrials.gov/ct2/show/NCT01336738?term=NCT01336738&draw=2&rank=1)/;2013 [access September 05, 2022].
11. Study of safety and efficacy of PF-04991532 in subjects with type 2 diabetes. [https://clinicaltrials.gov/ct2/show/NCT01338870?term=PF-04991532&draw=2&rank=1](https://clinicaltrials.gov/ct2/show/NCT01338870?term=NCT01338870&draw=2&rank=1)/;2013 [access September 05, 2022].
12. Vella, A. Freeman JLR, Dunn I, et al. Targeting hepatic glucokinase to treat diabetes with TTP399, a hepatoselective glucokinase activator. *Sci Transl Med.* 2019;**11**(475):eaau3441. https://doi.org/10.1126/scitranslmed.aau3441.
13. Klein KR, Freeman JLR, Dunn I, et al. The SimpliciT1 Study: a randomized, double-blind, placebo-controlled phase 1b/2 adaptive study of TTP399, a hepatoselective glucokinase activator, for adjunctive treatment of type 1 diabetes. *Diabetes Care.* 2021;**44**(4):960-968.

Table S3. Risk of bias of included studies

| Author, year | Adequate randomization sequence generation | Adequate allocation concealment | Blinding  of participants and caregivers | Blinding of  outcome assessors  and adjudicators | Free of incomplete outcome data | Free of selective outcome reporting | Free of other bias |  |
| --- | --- | --- | --- | --- | --- | --- | --- | --- |
| AZD1656 versus placebo | | | | | | | | |
| Kiyosue, 2013 | **Definitely yes**  Using computer generated randomisation list | **Definitely yes**  Interactive web-based response system | **Definitely yes** | **Definitely yes** | **Probably no**  There were 11.83% (20/169) and 9.09% (5/55) patients in AZD1656 and placebo groups with missing outcome data, respectively; missing outcome data were generally balanced across treatment groups, with similar reasons for missing data | **Definitely yes** | **Probably yes**  Baseline characteristics were generally balanced |  |
| Wilding, 2013 | **Definitely yes**  Using a randomization scheme generated by AstraZeneca | **Probably yes**  Randomized, double-blinded | **Definitely yes** | **Definitely yes** | **Probably no**  There were 16.67% (46/276) and 12.5% (11/88) patients in AZD1656 and placebo groups with missing outcome data, respectively; missing outcome data were generally balanced across treatment groups, with similar reasons for missing data | **Definitely yes** | **Probably yes**  Baseline characteristics were generally balanced |  |
| dorzagliatin versus placebo | | | | | | | | |
| Yang,2022 | **Definitely yes**  Using a permuted block randomization algorithm | **Definitely yes**  Interactive web-based response system | **Definitely yes** | **Definitely yes** | **Probably no**  There were 8.64% (33/382) and 10.9% (42/385) patients in dorzagliatin and placebo groups with missing outcome data, respectively; missing outcome data were generally balanced across treatment groups, with similar reasons for missing data | **Definitely yes** | **Probably yes**  Baseline characteristics were generally balanced |  |
| Zhu,2018 | **Definitely yes**  Using a permuted-block  schedule generated by an independent bio statistician using SAS PROC PLAN procedure | **Probably yes**  Randomized, double-blinded | **Definitely yes** | **Definitely yes** | **Probably no**  There were 13.66% (28/205) and 3.77% (2/53) patients in dorzagliatin and placebo groups with missing outcome data, respectively; missing outcome data were generally balanced across treatment groups, with similar reasons for missing data | **Definitely yes** | **Probably yes**  Baseline characteristics were generally balanced |  |
| Zhu,2022 | **Definitely yes**  Using a permuted block randomization algorithm | **Definitely yes**  Interactive web-based response system | **Definitely yes** | **Definitely yes** | **Probably no**  There were 9.35 % (29/310) and 21.57% (33/153) patients in dorzagliatin and placebo groups with missing outcome data, respectively; missing outcome data were generally balanced across treatment groups, with similar reasons for missing data | **Definitely yes** | **Probably yes**  Baseline characteristics were generally balanced |  |
| MK-0941 versus placebo | | | | | | | | |
| Meininger,2011 | **Probably yes**  Randomized, double-blinded | **Probably yes**  Randomized, double-blinded | **Definitely yes** | **Definitely yes** | **Probably no**  There were 6.36% (15/236) and 10.43% (12/115) patients in MK-0941 and placebo groups with missing outcome data, respectively; missing outcome data were generally balanced across treatment groups, with similar reasons for missing data | **Definitely yes** | **Probably yes**  Baseline characteristics were generally balanced |  |
| NCT00824616,2015 | **Probably yes**  Randomized, double-blinded | **Probably yes**  Randomized, double-blinded | **Definitely yes** | **Definitely yes** | **Probably no**  There were 20.59 % (7/34) and 17.64% (6/34) patients in MK-0941 and placebo groups with missing outcome data, respectively; missing outcome data were generally balanced across treatment groups, with similar reasons for missing data | **Definitely yes** | **Probably yes**  Baseline characteristics were generally balanced |  |
| PF-04937319 versus placebo | | | | | | | | |
| NCT01475461,2017 | **Probably yes**  Randomized, double-blinded | **Probably yes**  Randomized, double-blinded | **Definitely yes** | **Definitely yes** | **Probably no**  There were 9.42 % (21/223) and 15.79% (9/57) patients in PF-04937319 and placebo groups with missing outcome data, respectively; missing outcome data were generally balanced across treatment groups, with similar reasons for missing data | **Definitely yes** | **Probably yes**  Baseline characteristics were generally balanced |  |
| NCT01517373,2017 | **Probably yes**  Randomized, single-blinded | **Probably yes**  Randomized, single-blinded | **Definitely yes** | **Definitely yes** | **Probably no**  There were 10.44 % (19/182) and 6.56% (4/61) patients in PF-04937319 and placebo groups with missing outcome data, respectively; missing outcome data were generally balanced across treatment groups, with similar reasons for missing data | **Definitely yes** | **Probably yes**  Baseline characteristics were generally balanced |  |
| PF-04991532 versus placebo | | | | | | | | |
| NCT01336738,2013 | **Probably yes**  Randomized, double-blinded | **Probably yes**  Randomized, double-blinded | **Definitely yes** | **Definitely yes** | **Probably no**  There were 15.09% (24/159) and 18.87% (10/53) patients in PF-04991532 and placebo groups with missing outcome data, respectively; missing outcome data were generally balanced across treatment groups, with similar reasons for missing data | **Definitely yes** | **Probably yes**  Baseline characteristics were generally balanced |  |
| NCT01338870,2013 | **Probably yes**  Randomized, double-blinded | **Probably yes**  Randomized, double-blinded | **Definitely yes** | **Definitely yes** | **Probably no**  There were 18.91 % (38/201) and 18% (9/50) patients in PF-04991532 and placebo groups with missing outcome data, respectively; missing outcome data were generally balanced across treatment groups, with similar reasons for missing data | **Definitely yes** | **Probably yes**  Baseline characteristics were generally balanced |  |
| TTP399 versus placebo | | | | | | | | |
| Vella,2019 | **Probably yes**  Randomized, double-blinded | **Probably yes**  Randomized, double-blinded | **Definitely yes** | **Definitely yes** | **Definitely yes**  There were 1.08 % (1/93) and 0% (0/48) patients in TTP399 and placebo groups with missing outcome data, respectively; missing outcome data were generally balanced across treatment groups, with similar reasons for missing data | **Definitely yes** | **Probably yes**  Baseline characteristics were generally balanced |  |
| Klein,2021 | **Probably yes**  Randomized, double-blinded | **Probably yes**  Randomized, double-blinded | **Definitely yes** | **Definitely yes** | **Probably yes**  There were 6.12 % (3/49) and 3.57% (2/56) patients in TTP399 and placebo groups with missing outcome data, respectively; missing outcome data were generally balanced across treatment groups, with similar reasons for missing data | **Definitely yes** | **Probably yes**  Baseline characteristics were generally balanced |  |

Table S4. FINS, HOMA-β and HOMA-IR change from baseline with GKAs treatment versus placebo

|  |  | No. of studies | WMD  (μIU/mL) | 95% CI  (μIU/mL) | | P value | *I*^2^ (%) | Predictive Interval |
| --- | --- | --- | --- | --- | --- | --- | --- | --- |
| FINS change from baseline versus placebo | | | | | | |  |  |
| Total |  | 5 | -0.964 | -1.317 | -0.611 | ＜0.001 | 9.0 | -1.58 to -0.34 |
| Stratified by duration of follow-up | ＞12 weeks | 3 | -0.942 | -1.483 | -0.400 | 0.001 | 0.0 | -1.71 to -0.17 |
|  | =12 weeks | 2 | -0.981 | -1.447 | -0.514 | ＜0.001 | 42.6 | -2.57 to 0.65 |
| Stratified by duration of follow-up | ≥24 weeks | 2 | -0.048 | -3.397 | 3.300 | 0.977 | 22.0 | -21.76 to 21.66 |
|  | ＜24 weeks | 3 | -0.974 | -1.130 | -0.619 | ＜0.001 | 0.0 | -1.82 to -0.12 |
| Stratified by selectivity | Dual acting | 4 | -0.981 | -1.136 | -0.626 | ＜0.001 | 17.9 | -1.77 to -0.19 |
|  | Hepatoselective | 1 | 0.909 | -2.814 | 4.632 | 0.632 | 0.0 | / |
| Stratified by agent type | AZD1656 | 1 | -0.966 | -1.514 | -0.417 | 0.001 | 0.0 | -4.52 to 2.59 |
|  | Dorzagliatin | 1 | -4.100 | -11.760 | 3.560 | 0.294 | / | / |
|  | PB-201 | 2 | -0.981 | -1.447 | -0.514 | ＜0.001 | 42.6 | -2.57 to 0.65 |
|  | TTP399 | 1 | 0.909 | -2.814 | 4.632 | 0.632 | 0.0 | / |
| HOMA IR | Dorzagliatin | 3 | -0.077 | -0.133 | -0.022 | 0.006 | 0.0 | -0.44, 0.28 |
| HOMA-β | Dorzagliatin | 2 | 2.681 | 1.141 | 4.221 | 0.001 | 0.0 | / |

Abbreviations: FINS: fasting insulin; HOMA-IR: homeostasis model assessment of insulin resistance; HOMA-β: homeostasis model assessment-β; WMD: weighted mean difference; CI: confidence interval.

Table S5. Metabolic and safety indicators with GKAs treatment versus placebo in type 2 diabetes.

|  | No. of studies | Participants  (GKA group/PBO group) | WMD | 95% CI |  | P value | *I*^2^ (%) | Predictive Interval |
| --- | --- | --- | --- | --- | --- | --- | --- | --- |
| TCHO (mmol/L) | 3 (2 with dorzagliatin, 1 with TPP399) | 784/634 | 0.136 | 0.057 | 0.214 | 0.001 | 0.0 | -0.04, 0.31 |
| LDL-C (mmol/L) | 3 (2 with dorzagliatin, 1 with TPP399) | 784/634 | -0.029 | -0.109 | 0.050 | 0.471 | 12.0 | -0.25, 0.19 |
| HDL-C (mmol/L) | 3 (2 with dorzagliatin, 1 with TPP399) | 784/634 | 0.008 | -0.023 | 0.038 | 0.631 | 44.6 | -0.10, 0.12 |
| SUA (μmol/L) | 2 (dorzagliatin) | 692/538 | 29.07 | 18.11 | 40.03 | ＜0.001 | 52.7 | / |
| eGFR (ml/min/1.73m^2^) | 2 (dorzagliatin) | 692/538 | 1.34 | -1.81 | 4.49 | 0.400 | 77.7 | / |

Abbreviations: LDL-C: low-density lipoprotein cholesterol; HDL-C: high-density lipoprotein cholesterol; SUA: serum uric acid; eGFR estimated glomerular filtration rate; WMD: weighted mean difference; CI: confidence interval.

Table S6. Metabolic and safety indicators with GKAs treatment versus placebo in type 1 diabetes.

|  | No. of studies | Participants  (GKA group/PBO group) | WMD | 95% CI |  | P value |
| --- | --- | --- | --- | --- | --- | --- |
| TG (mmol/L) | 1 study with 2 parts | 46/54 | 0.036 | -0.355 | 0.427 | 0.857 |
| TCHO (mmol/L) | 1 study with 2 parts | 46/54 | -0.104 | -0.661 | 0.453 | 0.714 |
| LDL-C (mmol/L) | 1 study with 2 parts | 46/54 | -0.240 | -0.527 | 0.048 | 0.102 |
| HDL-C (mmol/L) | 1 study with 2 parts | 46/54 | 0.018 | -0.196 | 0.232 | 0.869 |

Abbreviations: LDL-C: low-density lipoprotein cholesterol; HDL-C: high-density lipoprotein cholesterol; WMD: weighted mean difference; CI: confidence interval.

Table S7. Meta-regression analyses for the associated factors with HbA1c change

|  | Coefficient | 95% CI |  | P value |
| --- | --- | --- | --- | --- |
| Age | 0.029 | -0.095 | 0.151 | 0.620 |
| Gender | 0.013 | -0.003 | 0.028 | 0.113 |
| Disease duration | -0.0181 | -0.073 | 0.037 | 0.482 |
| Baseline BMI | 0.013 | -0.063 | 0.088 | 0.370 |
| Baseline HbA1c | -0.151 | -0.771 | 0.473 | 0.602 |
| FPG change | 0.345 | 0.072 | 0.618 | 0.018 |
| TG change | -0.851 | -2.546 | 0.844 | 0.236 |

Abbreviations: HbA1c: hemoglobin A1c; FPG: fasting plasma glucose; BMI: body mass index; CI: confidence interval.

Table S8. Meta-regression analyses for the associated factors with TG change

|  | Coefficient | 95% CI |  | P value |
| --- | --- | --- | --- | --- |
| Age | -0.076 | -0.197 | 0.045 | 0.157 |
| Gender | 0.016 | -0.011 | 0.043 | 0.180 |
| Disease duration | 0.028 | -0.030 | 0.086 | 0.254 |
| Baseline BMI | -0.045 | -0.121 | 0.031 | 0.175 |
| Baseline HbA1c | 0.426 | -3.483 | 4.334 | 0.777 |
| HbA1c change | -0.377 | -1.635 | 0.881 | 0.452 |
| FPG change | -0.118 | -0.713 | 0.476 | 0.611 |

Abbreviations: HbA1c: hemoglobin A1c; FPG: fasting plasma glucose; BMI: body mass index; CI: confidence interval.

Table S9. Meta-regression analyses for the associated factors with risk of hypoglycemia

|  | Coefficient | 95% CI |  | P value |
| --- | --- | --- | --- | --- |
| Age | 0.175 | -0.497 | 0.846 | 0.571 |
| Gender | 0.029 | -0.072 | 0.130 | 0.537 |
| Disease duration | -0.290 | -0.692 | 0.112 | 0.137 |
| Baseline BMI | -0.113 | -0.583 | 0.357 | 0.600 |
| Baseline HbA1c | 1.832 | -1.062 | 4.727 | 0.186 |
| HbA1c change | -0.253 | -2.977 | 2.471 | 0.838 |
| FPG change | 0.052 | -0.746 | 0.850 | 0.886 |
| TG change | 4.819 | -2.114 | 11.752 | 0.140 |

Abbreviations: HbA1c: hemoglobin A1c; FPG: fasting plasma glucose; BMI: body mass index; CI: confidence interval.
